# High Charge Carrier Mobilities and Lifetimes in Organolead Trihalide Perovskites

**DOI:** 10.1002/adma.201305172

**Published:** 2013-12-20

**Authors:** Christian Wehrenfennig, Giles E Eperon, Michael B Johnston, Henry J Snaith, Laura M Herz

**Affiliations:** Oxford University, Clarendon LaboratoryParks Road, Oxford, OX1 3PU, UK

**Keywords:** CH_3_NH_3_PbI_3_, recombination, diffusion length, terahertz, photovoltaics

A new generation of thin-film photovoltaic cells based on organo-metal halide perovskite absorbers has recently emerged with extraordinary power conversion efficiencies (PCE).^1^ Initial PCEs reported around 10 % were soon superseded by values ranging from 12% to over 15% as materials control and device protocol improved.^2^ Methylammonium lead trihalide perovskite materials allow low-cost solution processing and absorb broadly across the solar spectrum, making them an exciting new component for clean energy generation. Interestingly, two different device concepts have appeared: in one, the lead halide compound CH_3_NH_3_PbI_3_ is deposited on the surface of mesoporous TiO_2_, which is then infiltrated with the solid-state hole transporter spiro-OMeTAD.[1a] The resulting cell has an analogous structure to a conventional solid-state dye-sensitized solar cell, in which upon photoexcitation electrons transfer from an excited dye to the TiO_2_ conduction band and subsequently diffuse to the contact.^3^ The first reported power conversion efficiencies of up to 9.7% for cells based on CH_3_NH_3_PbI_3_ by far exceeded previous records achieved with cells employing dyes as absorbers (≤6%).^4^ In another approach, Lee et al. obtained efficiencies of up to 10.9% using the mixed halide perovskite material CH_3_NH_3_PbI_3−x_Cl_x_ as both absorber and electron transporter, thus eliminating the need for a bulk heterojunction with a separate electron-transporting material. In this concept, the mesoporous TiO_2_ is replaced by Al_2_O_3_, which does not allow electron injection due to its high-lying conduction band, acting simply as an inert scaffold for the perovskite. Most recently, vapour deposition of highly uniform solid organolead trihalide perovskite films has been shown to allow PCEs as high as 15.4% in planar-heterojunction device structures.[2d]

Given the rapid rise in performance of organolead trihalide perovskite photovoltaic devices, little is known about what makes these materials so successful at generating and transporting photocurrents. Some work on transport properties has been published on the related tin halides^5^ and recently Hall-effect conductivity measurements have been performed on CH_3_NH_3_PbI_3_.^6^ Other studies on lead halides mostly focused on optical and excitonic properties^7^ or theoretical band structure calculations.^8^ It has recently been shown that charge carriers are capable of travelling over distances of up to a micron in some perovskite absorbers, which exceeds their typical absorption depth.^9^ However, the mechanism causing such an extended diffusion range remains mysterious, given that long charge-carrier diffusion distances require both low recombination rates and/or high charge mobility. Satisfying both requirements simultaneously is generally difficult given the fundamental Langevin limit for kinetic recombination, which typically holds for conductors with charge mobilities below the order of 1–10 cm^2^ V^−1^ s^−1^.^10^

In this work, we show that both CH_3_NH_3_PbI_3_ and CH_3_NH_3_PbI_3−x_Cl_x_ exhibit unexpectedly long charge carrier diffusion lengths because of non-Langevin charge carrier recombination. Using transient THz spectroscopy, we determine that bi-molecular recombination rates are abnormally low in these materials, defying the Langevin limit by at least four orders of magnitude. We also establish lower bounds for the high-frequency charge mobility of 11.6 cm^2^ V^−1^ s^−1^ for CH_3_NH_3_PbI_3−x_Cl_x_ and 8 cm^2^ V^−1^ s^−1^ for CH_3_NH_3_PbI_3_, which are remarkably high for solution-processed materials. The combination of high charge mobility and low bi-molecular recombination leads to carrier diffusion lengths that exceed one micron and are significantly longer for the mixed halide system. We propose that such reduced bi-molecular charge recombination arises from spatial separation of electrons and holes in the system, which may be tuned through substitutions affecting the electronic structure of the metal-halide system.

In order to highlight the relevance of our spectroscopic study to solar cell operation we correlated our findings with the performance of matching devices. **Figure** **1** a shows a schematic of the device structure employed: the metal-halide perovskite acts as both light absorber and charge transporter while spiro-OMeTAD functions as the hole transporter. The organolead trihalide perovskite is infiltrated into a 400 nm-thick scaffold layer of Al_2_O_3_ nanoparticles whose main function is to improve uniformity of coating, inhibiting pin-hole formation and the associated dark current leakage.^2^,^11^ We term this device a meso-superstructured solar cell (MSSC); however, it is important to note that electrons and holes have to travel through the perovskite layer to reach the respective extraction layers, compact TiO_2_ and spiro-OMeTAD, in analogy to a planar-heterojunction device structure. For spectroscopic measurements, metal-halide perovskite films were deposited under identical conditions on z-cut quartz, but with the electrodes and spiro-OMeTAD layers omitted.

**Figure 1:**
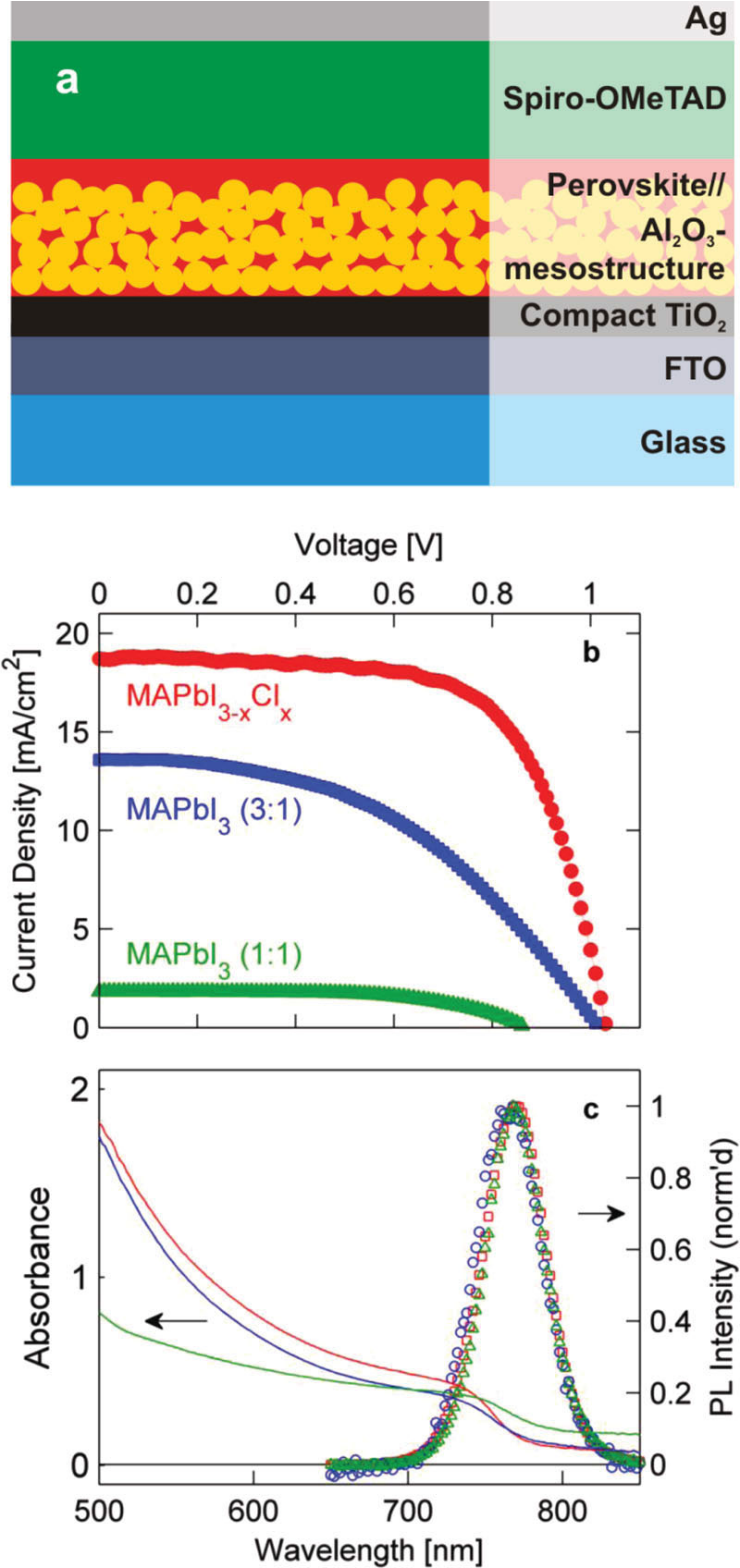
a) Diagrammatic representation of photovoltaic device structure. A 400 nm-thick layer of organolead trihalide perovskite on an Al_2_O_3_ nanoparticle scaffold acts as absorber and electron transporter, while a ∼350 nm thick layer of spiro-OMeTAD is used as hole conductor. b) Current density—voltage characteristics of photovoltaic devices employing triiodide (CH_3_NH_3_PbI_3_) and mixed halide (CH_3_NH_3_PbI_3−x_Cl_x_) lead perovskite absorbers, recorded under 1.5 AM simulated sunlight. Full device performance statistics are given in SI. c) Absorbance spectra of thin films of CH_3_NH_3_PbI_3−x_Cl_x_ (red line) CH_3_NH_3_PbI_3_ (3:1) (blue line) and CH_3_NH_3_PbI_3_ (1:1) (green line) together with photoluminescence spectra collected following excitation at 510 nm (CH_3_NH_3_PbI_3−x_Cl_x_ (red squares) CH_3_NH_3_PbI_3_ (3:1) (blue circles) and CH_3_NH_3_PbI_3_ (1:1) (green triangles)).

The mixed halide perovskite CH_3_NH_3_PbI_3−x_Cl_x_ was synthesized as described in[1b] and[2b] with excess methylammonium which may compensate losses of the organic material during the annealing process. The triiodide perovskite CH_3_NH_3_PbI_3_ reported in[1a] and[2a] on the other hand was synthesized at a 1:1 molar ratio of CH_3_NH_3_I to PbI_2_. For best comparability we therefore prepared two kinds of CH_3_NH_3_PbI_3_ materials – the non-stoichiometric type with excess methylammonium (3:1) and the stoichiometric type (1:1). Spectroscopy samples and devices were made using the same stock of perovskite precursor – full details can be found in Supporting Information.

Figure 1b presents typical current-density-voltage-characteristics of solar cells incorporating the three different organolead trihalide perovskites. Maximum power conversion efficiencies (PCE) recorded under simulated sunlight (AM1.5) were 12.7% for the mixed halide, 8.5 % for the non- stoichiometric (3:1) triiodide, and below 1% for the stoichiometric (1:1) triiodide perovskite. Detailed statistics of photovoltaic performance data for all fabricated devices can be found in SI. The low performance of devices incorporating CH_3_NH_3_PbI_3_ (1:1) may in part be attributed to poor film formation as evidenced by the large amount of scattering seen in the corresponding UV/vis absorption spectrum (Figure 1c).

We proceed to unravel the charge dynamics in the perovskite materials following pulsed photoexcitation. It is still unclear to what extent primary photoexcited species in organolead trihalide perovskites at room temperature divide into free charges or exciton populations. Exciton binding energies of between 37 meV^12^ and 50 meV[7d] have been proposed for (orthorhombic) CH_3_NH_3_PbI_3_ at low temperature (5 K). In both studies the diamagnetic shift of the magnetoabsoption spectrum was measured to magnetic fields in excess of 40 T and the exciton binding energy was derived making assumptions about the value of the high frequency dielectric function.^12^ We find that emission spectra and absorption onsets (Figure 1c) for all three investigated metal-halide perovskites are very similar, therefore any variation in exciton binding energy is likely to be relatively small. The proposed exciton binding energies of 37–50 meV are comparable to thermal energies at room temperature *k_B_T* ≈ 26 meV. Following photoexcitation, both free charge carriers and excitons may therefore be present, with their populations interchanging dynamically over the course of their lifetime.

**Figure** **2** displays the transient THz transmission dynamics following photoexcitation of CH_3_NH_3_PbI_3−x_Cl_x_ and CH_3_NH_3_PbI_3_ (3:1) for a range of different excitation fluences (data for CH_3_NH_3_PbI_3_ (1:1) in SI). In these experiments, the sample is excited by an optical light pulse at a wavelength of 550 nm and subsequently probed by a terahertz-frequency pulse after a well defined delay (see^13^ and SI for details of apparatus). In the presence of free charges, the measured relative change in THz electric field transmission is proportional to the photoinduced conductivity in the material.^14^ By scanning the full waveform of a THz pulse transmitted through the sample the complex dielectric function in the THz range is also directly reconstructed (THz spectra given in SI). In principle, any photoexcited species coupling to electro-magnetic THz radiation may be responsible for the observed THz transmission transients. We argue here that the observed dynamics arise solely from the photoconductivity of free charges. First, for an assumed exciton binding energy of ∼40 meV, the energy of probe photons employed here (1 THz corresponds to 4 meV) is too low to couple to significant intra-excitonic resonances. Second, the shape of the transient THz spectra is incompatible with an excitonic absorption and more akin to those reported to describe charge motion in polycrystalline materials (see SI).[14d] Third, we show below that the shape of the fluence-dependent transients is incompatible with a significant mono-molecular decay contribution from geminate recombination of excitons. We note however that we do not rule out a presence of excitons in the materials following photoexcitation; they simply do not appear to couple effectively to the THz probe.

**Figure 2:**
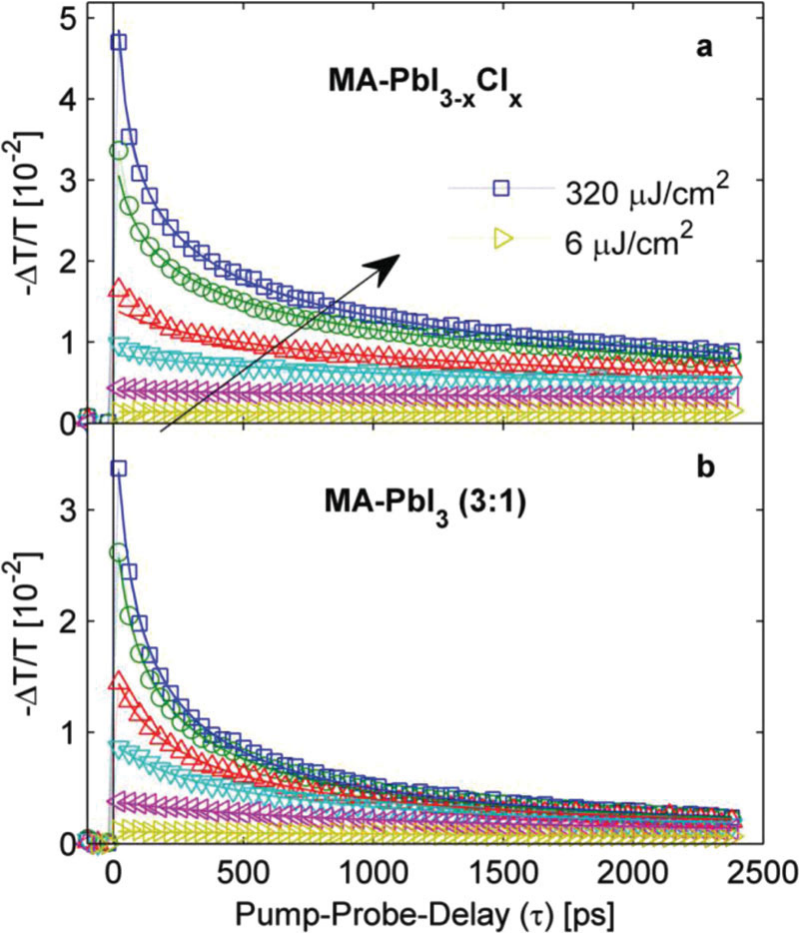
THz photoinduced absorption transient of a) CH_3_NH_3_PbI_3−x_Cl_x_ and b) CH_3_NH_3_PbI_3_ (3:1) after excitation at 550 nm for fluences between 6 μJ cm^−2^ and 320 μJ cm^−2^. Solid lines are fits based on second- and third-order charge recombination as described in the text and in SI.

Figure 2 reveals striking differences in the photoconductivity decay dynamics between the mixed halide and triiodide perovskites. Both CH_3_NH_3_PbI_3−x_Cl_x_ and CH_3_NH_3_PbI_3_ (3:1) show a fast initial decay component that becomes increasingly significant with increasing excitation fluence. However, for the triiodide perovskite, the fast component is much more pronounced at identical fluences (data for CH_3_NH_3_PbI_3_ (1:1) are very similar to those for CH_3_NH_3_PbI_3_ (3:1), see SI). The lowest fluence employed, 6 μJ cm^−2^, corresponds to an absorbed peak photon density of ∼6 × 10^17^ cm^−3^. At elevated charge carrier densities, higher-order effects such as bi-molecular (second order) and Auger (third order) charge recombination processes increasingly come into play. While bi-molecular recombination simply relies on an overlap of electron and hole wavefunctions, Auger processes involve energy and momentum transfer of the recombining electron-hole pair to a third charge carrier.^15^ To unravel the charge recombination rates associated with both mono-molecular (arising e.g. from trap-assisted recombination) and higher-order processes, we fit solutions to the differential equation

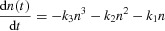
 to the THz photoconductivity transients, where *n* is the photoinduced charge carrier density. Here we assume that the photoconductivity decay is solely influenced by a change in charge carrier density, rather than mobility, which is reasonable given the absence of visible photoconductivity decay in the low fluence regime over the observation window (Figure 2a). Global fits were used for each set of fluence-dependent transients and the spatial variation of the charge density profile was taken into account (see SI for full details of fitting procedure). We make two striking observations from our analysis. First, we find that the monomolecular charge carrier recombination rate is exceptionally low in these materials. Data shown in Figure 2 allow for high-quality fits (solid lines) incorporating just second- and third-order decay components, which sets an upper limit of *k_1_* ≤ (25 ns)^−1^ (CH_3_NH_3_PbI_3−x_Cl_x_) and *k_1_* ≤ (6 ns)^−1^ (CH_3_NH_3_PbI_3_) for the monomolecular recombination rate. Such absence of trap- or impurity-assisted recombination over the timescale of nanoseconds is excellent news for use of organolead trihalide perovskites in photovoltaic cells and is in contrast with other materials commonly used, such as GaAs^16^ and mesoporous TiO_2_.^13^ Second, we find that the bi-molecular charge recombination rate (see **Table** **1**) extracted for the mixed halide perovskite is an order of magnitude lower than for triiodide materials. As we show below, this leads to markedly larger diffusion lengths even at elevated charge carrier densities for the mixed halide perovskite making this material more more suitable for planar-heterojunction devices.

**Table 1:** Charge-carrier decay constants, Langevin ratio, charge mobility and device efficiencies for organolead trihalide perovskite materials listed in Column 1. Columns 2 and 3 show the third (φ^2^*k_3_*) and second (φ*k_2_*) order charge-carrier decay constants as determined from fits to the excitation-fluence dependence of the THz transient photoconductivity data (Figure 2). Column 4 gives mono-molecular recombination rates *k_1_* derived from mono-exponential fits to the tails of photoluminescence decay transients taken under low-fluence excitation (see SI). Column 5 lists the effective charge carrier mobilities φμ derived from the initial THz photoconductivity. Column 6 compares the bimolecular-recombination-rate-to-mobility ratio (*k_2_*/μ)*_ex_* (from Columns 3 and 5) to that expected from Langevin theory (*k_2_*/μ)*_L_* = *e* (ε_0_ε*_r_*)^−1^, multiplied by ε*_r_*^−1^. Column 7 lists maximum power conversion efficiencies achieved with the material incorporated into full photovoltaic device structures

Material	Charge Carrier Decay Constants			Mobilitya)	Langevinb)	Device
	3rd ordera) [cm^6^ s^−1^]	2nd ordera) [cm^3^ s^−1^]	1st order (PL) [μs^−1^]	φμ [cm^2^ V^−1^ s^−1^]	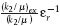	PCE_max_ [%]
CH_3_NH_3_PbI_3−x_Cl_x_	9.9 × 10^−29^	8.7 × 10^−11^	4.9	11.6	4.2 × 10^−6^	12.7
CH_3_NH_3_PbI_3_ (3:1)	3.7 × 10^−29^	9.4 × 10^−10^	15	8.1	6.4 × 10^−5^	8.5
CH_3_NH_3_PbI_3_ (1:1)	1.3 × 10^−28^	9.2 × 10^−10^	14	8.2	6.2 × 10^−5^	0.9

^a)^The branching ratio φ of generated free charge-carrier density per absorbed photon density is unknown, but falls in the range 0 ≤ φ ≤ 1. Hence values listed in Columns 2, 3, 4 represent lower limits of *k_2_* and *k_3_* and μ;

^b)^The possible range for ε*_r_* is limited to the interval between the electric permitivities of its consituents: Vacuum (ε*_r_* = 1), Al_2_O_3_ (ε*_r_* = 1.77)^17^ and CH_3_NH_3_PbI_3_ (ε*_r_* = 6.5)^12^

Performance differences between the three organolead trihalide perovskites could also potentially arise from discrepancies in the charge carrier mobility μ. We elucidate such effects, by determining the effective charge carrier mobility for the three organolead trihalide perovskites in the low-fluence regime. Such values can be directly derived from the photoinduced change of THz electric field transmission Δ*T*/*T* (as shown in Figure 2) which is proportional to the photoconductivity. We extract the effective charge carrier mobility φμ from the photoconductivity onset value (prior to charge recombination) with knowledge of the absorbed photon density and optical parameters, as described in SI. Here, φ is the ratio of free-charge-carrier density generated per photon density absorbed, which is unknown and may depend on factors such as the exciton binding energy. We determine effective mobilities of 11.6 cm^2^ V^−1^ s^−1^ for CH_3_NH_3_PbI_3−x_Cl_x_ and ∼8 cm^2^ V^−1^ s^−1^ for both CH_3_NH_3_PbI_3_ variants. These values are exceptionally high for solution-processed materials, surpassing charge mobilities reported for mesoporous TiO_2_ used in dye-sensitized solar cells by at least a factor of 20,^13^,^18^,^19^ and those of typical π-conjugated molecular semiconductors by several orders of magnitude.^19^ We note that effective charge carrier mobility values represent lower bounds for the actual mobilities since the photon-to-charge branching ratio φ must fall between 0 and 1. Interestingly, Stoumpos et al. recently reported a DC dark electron mobility of ∼66 cm^2^ V^−1^ s^−1^ for unintentionally doped single crystalline bulk CH_3_NH_3_PbI_3_ obtained from 4-probe resistivity and Hall-effect measurements.^6^ Comparison with our values suggests that, within the nano-second diffusion range, the solution-processed materials investigated here do not incur substantial charge carrier mobility losses at grain boundaries. We stress that effective charge carrier mobilities are relatively similar for the mixed halide and trihalide perovskite materials. Given that the emission spectra for the two types of materials suggest similar exciton binding energies, φ is likely to vary little, and hence the cause in the differences in planar-heterojunction PV performance is unlikely to arise from differences in charge carrier mobility. We therefore propose that the improved performance of the mixed halide in perovskite MSSCs originates from lower charge recombination rates, rather than better charge mobility.

Table1 summarizes the second and third-order recombination constants and effective mobilities determined for the three organolead trihalide perovskite materials. From these values, we may compute the ratio, *k_2_*/μ, of the bi-molecular recombination constant to the charge carrier mobility and compare it to the value expected from Langevin theory, *e* (ε*_0_*ε*_r_*)^−1^, where *e* is the elementary charge and ε*_r_* the appropriate value of the dielectric function.^10^ Remarkably, all three organolead trihalide perovskite materials defy the Langevin recombination limit by at least 4 orders of magnitude (see Table1). The Langevin model is based on a purely kinetic approach assuming that recombination will occur once an electron and a hole move within their joint capture radius, which is presumed to be larger than their mean free path.^10^ Materials whose ratio of *k_2_*/μ falls substantially short of the Langevin limit are naturally highly suitable for use in photovoltaics, allowing a low charge recombination rate without the penalty of lowered charge mobility. We note that charge recombination rates beyond the Langevin limit have been observed in other low-mobility materials that have been successfully incorporated in photovoltaic cells, such as solution-processed de-mixed blends of conjugated polymer with fullerene derivatives^19^,^20^ and amorphous silicon.^21^ In the former, such effects have been attributed to electrons and holes being separated into two material components, while in the latter, spatial charge separation through a random potential landscape has been postulated. As we discuss below, the metal-halide electronic structure may induce an intrinsic spatial charge separation that reduces recombination rates.

To illustrate the relative contributions to the overall charge decay rate (*R_total_*)*_,_* **Figure** **3**(a,b) shows the first-, second- and third order electron-hole recombination rates as a function of charge carrier density in the medium. Here, the mono-molecular recombination rate *k_1_* was determined from time-resolved photoluminescence transients taken at ultra-low excitation fluences (to avoid the dominance of higher-order effects) over the time scale of a few hundred nanoseconds (see SI). As already indicated by the THz transients, we find exceptionally low mono-molecular rates (Table1) for all organolead trihalide perovskites, which range between 5 and 15 μs^−1^. However, in contrast to the THz response, the PL emitted from the sample may originate from both excitonic and free-charge species, and hence mono-molecular PL lifetimes may be governed by both geminate recombination of excitons and trap-assisted charge recombination. Figure 3(a,b) shows that both mono- and bi-molecular recombination make dominant contributions at effective charge carrier densities of *n*φ = 10^15^ – 10^17^ cm^−3^, but the associated rate constants (*k_1_* and *k_2_*) are significantly lower for the mixed halide than the triiodide perovskite under typical device operating conditions. We extract a diffusion length *L* from these trends using *L*(*n*) *=* (*D*/*R_total_*(*n*))^1/2^, where *D* = μ*k_B_T e*^−1^ is the diffusion constant. Figure 3(c) displays the dependence of the diffusion length on charge carrier density derived for the cases of a photon to charge branching ratio of 1, corresponding to THz mobilities of 11.6 cm^2^ V^−1^ s^−1^ and 8.1 cm^2^ V^−1^ s^−1^ for CH_3_NH_3_PbI_3−x_Cl_x_ and CH_3_NH_3_PbI_3_ (3:1), respectively. We note that for φ < 1 diffusion lengths would be even larger. We find that substantial diffusion lengths in the micron-range are achievable for low charge carrier densities, in agreement with recent measurements based on diffusive exciton quenching.^9^ In accordance with device performance, the mixed-halide perovskite exhibits a diffusion length that is up to a factor four larger than that of the triiodide perovskite over charge carrier densities corresponding to realistic device operating conditions. In the presence of long range structural disorder, diffusion lengths may be somewhat shorter, as THz conductivity probes sample the material mobility on shorter length scales that may not include full conduction pathways to electrodes.^22^ Such extended charge diffusion lengths are to be expected, given that planar-heterojunction devices have now been realized,[2d] which require diffusion lengths to be comparable to or exceed optical absorption depths (typically 445 nm at 700 nm excitation and 120 nm at 500 nm excitation wavelength). Here we show that the reason for the long charge diffusion lengths is that organolead trihalide perovskites defy the Langevin limit. If bi-molecular recombination were as fast as derived from a kinetic model (Langevin theory), charge diffusion lengths would be reduced to the tens of nanometer range necessitating the use of a bulk heterojunction (mesostructured) device layout. These findings therefore show that the observed combination of low charge carrier recombination and high mobility is crucial to the development of planar heterojunction photovoltaic devices incorporating organolead trihalide perovskites.

**Figure 3:**
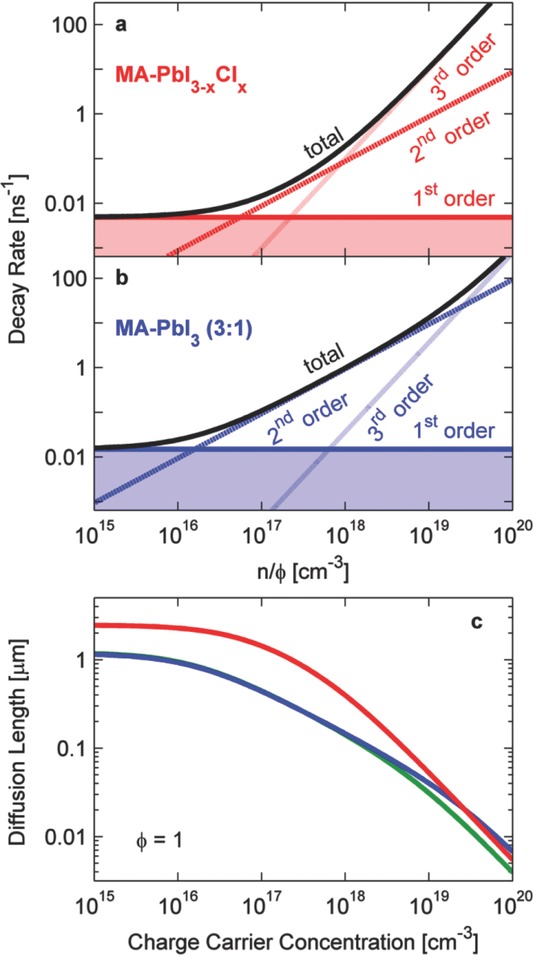
Charge-carrier decay rates and diffusion lengths of organolead trihalide perovskites as a function of charge carrier concentration, extracted from analysis of transient photoconductivity and photoluminescence measurements. a,b) Charge-carrier decay rates for CH_3_NH_3_PbI_3−x_Cl_x_ and CH_3_NH_3_PbI_3_ (3:1) as a function of *n*φ, where *n* is the charge carrier density in the materials and φ the ratio of *n* to the absorbed photon density. Rates are computed from the rate constants determined in this work (see Table1). Plotted are the 1st (monomolecular, solid), 2nd (bimolecular, dashed) and 3rd order (Auger, dotted) contribution to the total decay rate (black, solid), where *R*_1_ = *k*_1_, *R*_2_ = φ *k*_2_ (*n*/φ), *R*_3_ = φ^2^*k*_3_ (*n*/φ)^2^ and *R*_total_ = d*n*/(*n*d*t*) is the sum total. c) Charge-carrier diffusion lengths *L* = (μ*k_B_T*/(*e R_total_*))^1/2^ for CH_3_NH_3_PbI_3−x_Cl_x_ (red), CH_3_NH_3_PbI_3_ (3:1) (blue) and CH_3_NH_3_PbI_3_ (1:1) (green) as a function of charge-carrier concentration *n*, derived from charge-carrier decay rates and THz mobilities of 11.6 cm^2^ V^−1^ s^−1^, 8.1 cm^2^ V^−1^ s^−1^ and 8.2 cm^2^ V^−1^ s^−1^ (φ = 1).

To propose an explanation for these effects, we note that there are interesting parallels with early low-temperature measurements on lead-halide ionic crystals, which showed that while PbI_2_ exhibited sub-nanosecond PL lifetimes,^23^ the lighter-halide PbCl_2_^24^ exhibited much longer (microsecond) lifetimes. In some metal halide systems, such effects have been attributed to spatial localization of the hole in the halide vicinity.^25^ Clearly, in the organolead trihalide perovskites under investigation here, charges are found to be highly mobile and trapping mechanisms surprisingly absent. However, a weak preferential localization of electrons and holes in different regions of the perovskite unit cell may still result in a reduction in the spatial overlap of electron and hole wavefunctions and hence recombination rates. We note that density functional calculations on organolead triiodide perovskites have revealed that valence band maxima consist of 6s- and 5p orbitals of lead and iodine, respectively, while conduction band minima mostly incorporate 6p-orbitals of lead.[8d] This scenario is reminiscent of the case of metal alkali halides for which long life times had been reported earlier.^25^ Such spatial charge separation may explain the stark deviation from Langevin theory we observe for charge recombination in the organolead trihalide perovskites. Understanding and predictive modelling of such effects will therefore allow for directed photovoltaic material and device development.

In conclusion, we find that methylammonium lead trihalide perovskites are particularly well-suited as light absorbers and charge transporters in photovoltaic cells because they allow for an unexpected combination of both low charge recombination rates and high charge-carrier mobilities. We establish lower bounds of ∼10 cm^2^ V^−1^ s^−1^ for the high-frequency charge mobility, which is remarkably high for a solution-processed material. We reveal that planar heterojunction photovoltaic cells may only be achieved because the ratio of bi-molecular charge recombination rate to charge mobility is over four orders of magnitude lower than that predicted from Langevin theory. Such effects are likely to arise from spatial separation of opposite charge carriers within the metal-halide structure or across a crystalline domain. Modelling and tuning recombination channels, e.g. through halide and metal substitutions, or crystallite size, will hold the clue to raising material performance.

## Supporting Information

As a service to our authors and readers, this journal provides supporting information supplied by the authors. Such materials are peer reviewed and may be re-organized for online delivery, but are not copy-edited or typeset. Technical support issues arising from supporting information (other than missing files) should be addressed to the authors.
