# Supplementary material for: High Charge Carrier Mobilities and Lifetimes in Organolead Trihalide Perovskites
Source: Adv Mater. 2013 Dec 20;26(10):1584–9. doi: 10.1002/adma.201305172 (PMC4722848; doi:10.1002/adma.201305172)
Supplement: Supplementary file 1 — Supplementary [file adma0026-1584-sd1.pdf]

Copyright WILEY-VCH Verlag GmbH & Co. KGaA, 69469 Weinheim, Germany, 2013.

ADVANCED MATERIALS

Supporting Information

for *Adv. Mater.*, DOI: 10.1002/adma.201305172

High Charge Carrier Mobilities and Lifetimes in Organolead Trihalide Perovskites

*Christian Wehrenfennig, Giles E. Eperon, Michael B. Johnston, Henry J. Snaith, and Laura M. Herz**

Copyright WILEY-VCH Verlag GmbH & Co. KGaA, 69469 Weinheim, Germany, 2013.

Supporting Information

for *Adv. Mater.*, DOI: 10.1002/adma.201305172

High Charge Carrier Mobilities and Lifetimes in Organo Lead Trihalide Perovskites

*Christian Wehrenfennig, Giles E. Eperon, Michael B. Johnston, Henry J. Snaith, and Laura M. Herz**

A. Materials and Device Preparation

Perovskite Precursor Preparation: Methylammonium iodide (MAI) was prepared by reacting methylamine, 33 wt% in ethanol (Sigma-Aldrich), with hydroiodic acid (HI) 57 wt% in water (Sigma-Aldrich), at room temperature. HI was added dropwise while stirring. Upon drying at 100°C, a white powder was formed, which was dried overnight in an open dish on a hotplate before use. To form the non-stoichiometric $CH_3NH_3PbI_{3-x}Cl_x$ precursor solution, methylammonium iodide and lead (II) chloride (Sigma-Aldrich) are dissolved in anhydrous N,N-Dimethylformamide (DMF), at a 3:1 molar ratio of MAI to $PbCl_2$, with final concentrations 0.88 M lead chloride and 2.64 M methylammonium iodide. This solution is stored under a dry nitrogen atmosphere. For the stoichiometric (1:1) $CH_3NH_3PbI_3$ precursor solution MAI was dissolved in a 1:1 molar ratio with lead (II) iodide (Sigma-Aldrich) in DMF, with final concentrations 0.88 M and for the non-stoichiometric (3:1) $CH_3NH_3PbI_3$ precursor solution MAI was dissolved in a 3:1 molar ratio with lead (II) iodide (Sigma-Aldrich) in DMF, with final concentrations 0.88 M lead iodide and 2.64 M MAI.

Device substrates: Devices were fabricated on fluorine-doped tin oxide (FTO) coated glass (Pilkington TEC7, 7 Ω/sq). Initially FTO was removed from regions under the anode contact, to prevent shunting upon contact with measurement pins, by etching the FTO with 2 M HCl

and zinc powder. Substrates were then cleaned sequentially in 2 % hallmanex detergent, acetone, propan-2-ol and oxygen plasma. A hole-blocking layer of compact TiO_2 was deposited by spin-coating a mildly acidic solution of titanium isopropoxide in ethanol, and annealed at 500°C for 30 min.

Perovskite solar cells: A colloidal dispersion of <50 nm Al_2O_3 nanoparticles in isopropanol was spin-coated on the substrates followed by drying at 150°C. Then, to form the perovskite layer, the perovskite precursor solution was deposited and spin-coated at 2000 rpm. Films were then annealed at 100°C for 10 min ($CH_3NH_3PbI_3$ (1:1)), 150°C for 15 min ($CH_3NH_3PbI_3$ (3:1)), or 100°C for unit 45 min ($CH_3NH_3PbI_{3-x}Cl_x$). A hole-transporting layer was then deposited in air by spin-coating at 2000 rpm a 0.79 M solution in chlorobenzene of 2,2',7,7'-tetrakis-(N,N-di-p-methoxyphenylamine)9,9'-spirobifluorene (spiro-OMeTAD), with additives of lithium bis(tri-fluoro-methane-sulfonyl)imide and 4-tert-butylpyridine.[1] Devices were then left overnight in air for the spiro-OMeTAD to dope via oxidation. Finally, 60 nm gold electrodes were thermally evaporated under vacuum of $\sim 10^{-6}$ mbar, at a rate of ~ 0.1 nm s^{-1}, to complete the devices.

Samples for THz and PL experiments: Samples for THz and PL measurements consist of a layer of perovskite infiltrating a film of 500 nm mesoporous Al_2O_3. This layer was deposited by spin coating on 2 mm thick z-cut quartz in the same way as described above for the active layer of the solar cells.

B. Solar cell performance data

Solar Cell Characterization: The current density-voltage (J-V) curves were measured (2400 Series SourceMeter, Keithley Instruments) under simulated AM 1.5 simulated sunlight at 100 mW cm^{-2} irradiance generated by an Abet Class AAB sun 2000 simulator, with the intensity calibrated with an NREL calibrated KG5 filtered Si reference cell. The mismatch factor was calculated to be less than 1 %. The solar cells were masked with a metal aperture to define the active area, typically 0.09 cm^2 and measured in a light-tight sample holder to minimize any edge effects and ensure that the reference cell and test cell are located in the same spot under the solar simulator during measurements.

Table S1 lists detailed statistics of device performance for solar cells based on the three investigated perovskite absorbers, $CH_3NH_3PbI_{3-x}Cl_x$, non-stoichiometric $CH_3NH_3PbI_3$ and stoichiometric $CH_3NH_3PbI_3$. Minimum, maximum and average values for power conversion efficiency (PCE), short circuit current density j_{SC}, open circuit voltage V_{OC} and fill factor (FF) for all batches are summarized in the table.

Table S1. Device performance statistics for solar cell batches fabricated with the mixed halide and triiodide perovskites as absorbers on mesoporous Al_2O_3 scaffolds, fabricated as described in the main manuscript. The numbers of devices tested were: 44 ($CH_3NH_3PbI_{3-x}Cl_x$), 37 ($CH_3NH_3PbI_3$ (3:1)) and 26 ($CH_3NH_3PbI_3$ (1:1)).

Absorber Material		j_{SC} [mA cm^{-2}]	V_{OC} [V]	FF []	η [%]
	min	4.9	0.67	0.57	2.6
**$CH_3NH_3PbI_{3-x}Cl_x$**	max	18.9	1.05	0.68	12.7
	avg	12.6	0.98	0.64	8.0
	min	3.9	0.92	0.46	2.3
**$CH_3NH_3PbI_3$ (3:1)**	max	13.6	1.04	0.74	8.5
	avg	10.0	0.99	0.60	6.7
	min	0.5	0.79	0.34	0.2
**$CH_3NH_3PbI_3$ (1:1)**	max	1.9	0.91	0.72	0.9
	avg	0.86	0.86	0.56	0.5

C. Details of optical-pump THz-probe experiments

THz Time-Domain Spectroscopy: Our optical-pump-THz-probe setup (described in more detail previously[2]) uses a Ti:Sapphire regenerative amplifier to generate 40 fs pulses at 800 nm wavelength and a repetition rate of 1.1 kHz. Terahertz pulses are generated by optical rectification in 450 μm thick (110)-GaP and detected by electrooptic sampling in ZnTe crystal (0.2 mm (110)-ZnTe on 3 mm (101)-ZnTe). Pulses for optical excitation of the samples at a wavelength of 550 nm have been generated using an optical parametric amplifier (OPA). Optical excitation was carried out through the substrate side of the sample. The diameters of pump and probe beam at the sample position are 2.6 mm and 1.5 mm (FWHM). Measurements have been performed with the entire THz beam path (including emitter, detector and sample) in an evacuated chamber at pressure $p < 10^{-1}$ mbar.

D. Derivation of photoconductivity and charge carrier mobility from change in THz electric field transmission

The sheet photoconductivity ΔS of a thin film between two media of refractive indices n_A and n_B, under the condition that the thickness of the film is much smaller than the THz wavelength, may be expressed as[3,4b]

$$\Delta S = -\epsilon_0 c(n_A + n_B)\left(\frac{\Delta T}{T}\right), \qquad (1)$$

where $\Delta T = T_{illuminated} - T$ is the photoinduced change in terahertz electric field. Here, T and $T_{illuminated}$ are the transmitted terahertz electric fields of the sample in the dark and after photoexcitation respectively. Since in our samples the absorption depth is lower than the film thickness, the former marks the effective thickness of the photoexcited layer. Therefore, n_B is the refractive index of the alumina scaffold coated in perovskite (n_B = 1.5 ($CH_3NH_3PbI_{3-x}Cl_x$), 1.5 ($CH_3NH_3PbI_3$ (3:1)) and 1.65 ($CH_3NH_3PbI_3$ (1:1)) as determined from measurements of the phase delay of the THz wave with respect to a reference substrate). In the present work the thin film sample was deposited on a z-quartz substrate and excited through the substrate, hence $n_A = 2.13$ is the refractive index of z-cut quartz at THz frequencies.

In order to derive the charge-carrier mobility μ from the photoinduced sheet conductivity, the number of photo-excited charge carriers N needs to be determined using

$$N = \phi \frac{E\lambda}{hc}(1 - R_{pump})(1 - T_{pump}) \qquad (2)$$

Here, E is the energy contained in an optical excitation pulse of wavelength λ, R_{pump} is the reflectivity of the sample at normal incidence of the excitation beam, T_{pump} the (small) fraction of light transmitted through the sample and φ is the ratio of charges created per photons absorbed, commonly referred to as the photon-to-charge branching ratio, which is

undetermined in the experiment and related to factors such as the exciton binding energy in the material.

The charge carrier mobility μ is given by

$$\mu = \frac{\Delta S\, A_{eff}}{Ne} \tag{3}$$

where A_{eff} is the effective area of the overlap of optical pump and THz probe pulse taking into account the Gaussian beam profiles and e the elementary charge. Since φ is unknown, the quantity which can be directly derived from the experiment is the effective mobility $\tilde{\mu} = \phi\mu$ where

$$\phi\mu = -\varepsilon_0 c(n_A + n_B)\frac{A_{eff}hc}{E\, e\, \lambda\, (1 - R_{pump})(1 - T_{pump})}\left(\frac{\Delta T}{T}\right) \tag{4}$$

Because $0 \leq \varphi \leq 1$, the effective mobility represents a lower limit, which becomes identical to the actual mobility for full photon to free carrier conversion. The determined charge carrier mobility arises from the contributions of both electrons and holes, which cannot be separated. Therefore the extracted mobility value is the sum of electron and hole mobilities.

To allow accurate determination of φμ we ensured that excitation conditions are in the linear regime. We have evaluated the dependence of the initial amplitude of the THz response on excitation fluence and found that nonlinear processes such as two-photon absorption only show a significant effect at fluences beyond 60 μJ cm^{-2}. Our carrier mobility calculation is based on data taken at 6 μJ cm^{-2} and therefore not affected.

E. Fits to THz photoconductivity transients

As described in the manuscript, the recombination dynamics of the free charge carrier density n may be governed by mechanisms of first, second and third order, through the following rate equation:

$$\frac{\mathrm{d}n}{\mathrm{d}t} = -k_3 n^3 - k_2 n^2 - k_1 n\,, \tag{5}$$

As mentioned above, the photoinduced THz transmission change is proportional to the induced charge-carrier density which is related to the absorbed photon density $\tilde{n}$ through $n = \phi\tilde{n}$. Substituting into the above equation yields:

$$\frac{\mathrm{d}\tilde{n}}{\mathrm{d}t} = -k_3\phi^2\tilde{n}^3 - k_2\phi\tilde{n}^2 - k_1\tilde{n}\,, \tag{6}$$

Therefore, fitting the observed THz transients with decay mechanisms up to the third order under knowledge of the initially absorbed photon density, will yield the decay constants $k_3\varphi^2$, $k^2\varphi$ and k_1. To fit the THz transients displayed in Figure 2 of the main manuscript, $\mathrm{d}\tilde{n}/\mathrm{d}t$ was computed as a function of $\tilde{n}$ for all curves, and a third order polynomial was fitted to the joint dataset. A good reproduction of the data could be achieved even if the linear term was omitted. We hence proceeded to carry out full fits without the linear terms in order to determine the second- and third-order decay constants with high accuracy. For these fits, rate constants were only allowed to vary globally (i.e. had constant values across curves taken at all fluences). We verified that neglecting the first order contribution was actually justified by comparison to full fits with this component reinserted and established upper limits of values for k_1 of $(25\,\mathrm{ns})^{-1}$ for $CH_3NH_3PbI_{3-x}Cl_x$ and $(6\,\mathrm{ns})^{-1}$ for $CH_3NH_3PbI_3$ in agreement with visually flat curves observed for $CH_3NH_3PbI_{3-x}Cl_x$ in the low fluence regime over the 2.5 ns window.

To account for the spatially varying charge density profile, our fit routine takes into account the exponential charge density profile created by the pump beam by dividing the sample into

50 equally thick slabs and computing the decay function for all of these individually. Here, the initially absorbed photon density in the surface layer the pump is incident on is given by

$$\tilde{n}_0 = \frac{E\lambda\, \alpha(\lambda)}{hc\, A_{eff}}(1 - R_{pump}) \qquad (7)$$

where α is the absorption coefficient at the excitation wavelength.

In order to determine decay rates from fluence-dependent data, part of which were acquired under strong (nonlinear) excitation (up to 320 μJ cm^{-2}), it was necessary to extrapolate carrier densities from the linear (up to ~60 μJ cm^{-2}) to the nonlinear regime. This was required because of a somewhat sublinear dependence of the excited carrier concentration on fluence at strong excitation, which prevents that the ratio of the amplitudes is fixed at the ratio of excitation fluences. We therefore extrapolate the proportionality constant determined at low excitation fluence to determine the carrier densities at high fluences by scaling with the initial amplitude of the THz response.

F. Determination of charge-carrier diffusion length

From decay rates and mobilities we calculate the charge carrier diffusion length as a function of carrier density as follows:

$$L(n) = \sqrt{\frac{D}{R_{total}(n)}} = \sqrt{\frac{\mu\, k_B T}{e\, R_{total}}} \tag{8}$$

Here, $D = \mu k_B T e^{-1}$ is the diffusion constant at room temperature T as determined from the carrier mobility, and the total charge carrier recombination rate R_{total} can be determined from the decay constants listed in Table 1 of the main manuscript through

$$R_{total} = -\frac{1}{n}\frac{\mathrm{d}n}{\mathrm{d}t} = \phi^2 k_3 (n/\phi)^2 + \phi k_2 (n/\phi) + k_1 \equiv R_3 + R_2 + R_1 \tag{9}$$

R_{total}, R_3, R_2 and R_1 are plotted in Figure 3(a,b) of the main manuscript as a function of n/φ, while $L(n)$ is shown in Figure 3(c) as a function of n for the case of $\varphi = 1$.

G. THz photoconductivity transients for stoichiometric $CH_3NH_3PbI_3$

THz photoconductivity transients of $CH_3NH_3PbI_{3-x}Cl_x$ and non-stoichiometric $CH_3NH_3PbI_3$ across a wide range of excitation fluences were presented in Figure 2 in the main article. Here we show corresponding data for the stoichiometric triiodide perovskite $CH_3NH_3PbI_3$ (**Figure S1**). The data strongly resemble those from non-stoichiometric $CH_3NH_3PbI_3$ and fits yield very similar rate constants, as summarized in Table 1 of the main manuscript.

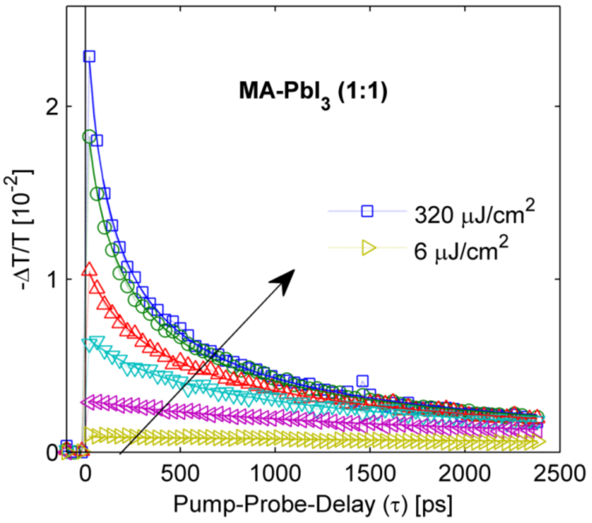

Figure S1. THz photoinduced absorption transient of stoichiometeric $CH_3NH_3PbI_3$ (see main text for $CH_3NH_3PbI_{3-x}Cl_x$ and non-stoichiometric $CH_3NH_3PbI_3$) for a range of fluences between 6 μJ cm^{-2} and 320 μJ cm^{-2} (Excitation wavelength: 550 nm). Fits: Decay function with a third and a second order component as described in the text.

H. THz photoconductivity spectra

THz photoconductivity spectra for $CH_3NH_3PbI_3$ and $CH_3NH_3PbI_{3-x}Cl_x$ are shown in **Figure S2** for pump-probe delays between 5 ps and 2 ns. Importantly for each sample there is no significant change in the spectra as a function of time after photoexcitation (i.e. no pump-probe delay dependence of the photoinduced phase shift of the transmitted THz pulse in the time domain). In this case carrier dynamics may be recorded by observing changes in the peak terahertz signal in the time domain as a function of pump-probe delay.[4c]

The shape of the transient THz photoconductivity spectra are consistent with the motion of photogenerated free charge exhibiting long-range scattering.[4,5] Over the measurable frequency range (0.2 – 3 THz) the real part of the conductivity $\propto \Delta T/T$ (see Equation 1) is offset from zero and relatively constant and the imaginary part of the conductivity is close to zero. This is consistent with a Drude conductivity, with relaxation rate $\gg$ 3 THz.[4b,c] In addition the features observable at 1 THz and 2 THz originate from a photoinduced modulation of LO-phonon modes superimposed on the Drude response. The LO-photon modes can be see in the absorption spectrum of the unilluminated samples, which are shown in **Figure S3**.

We also performed a control experiment showing that the shape of the THz spectrum is independent of the electric field strength of the THz pulse (see **Figure S4**), to confirm that the THz pulse was acting as a weak probe.

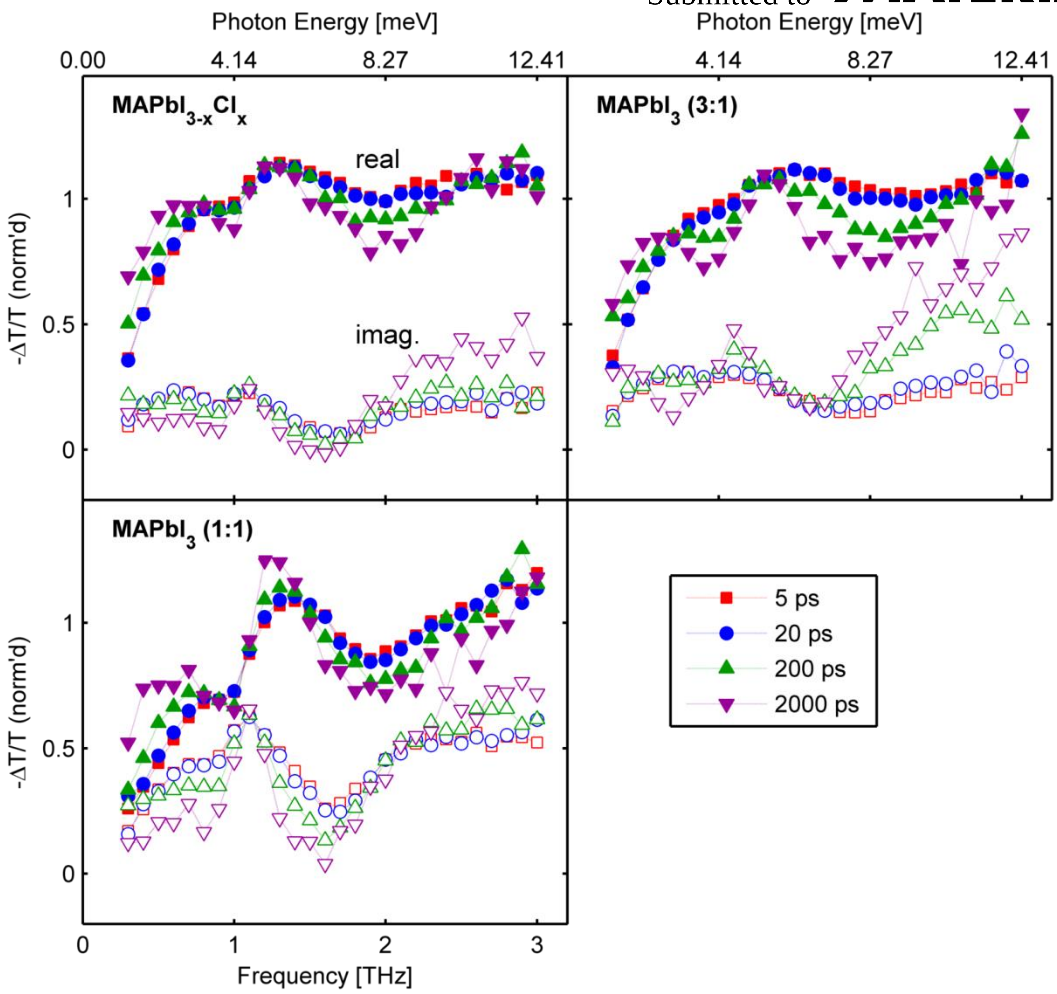

Figure S2. Photoinduced THz absorption spectra of $CH_3NH_3PbI_3$ and $CH_3NH_3PbI_{3-x}Cl_x$ at various times after excitation. (Excitation wavelength: 550 nm), fluence: 320 μJ cm^{-2}. For comparability, all spectra are normalized by their frequency-averaged absolute value.

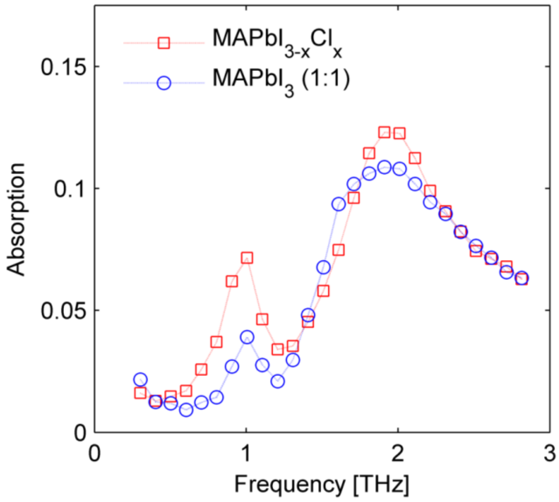

Figure S3. Dark THz absorption spectra of $CH_3NH_3PbI_{3-x}Cl_x$ and stoichiometeric $CH_3NH_3PbI_3$.

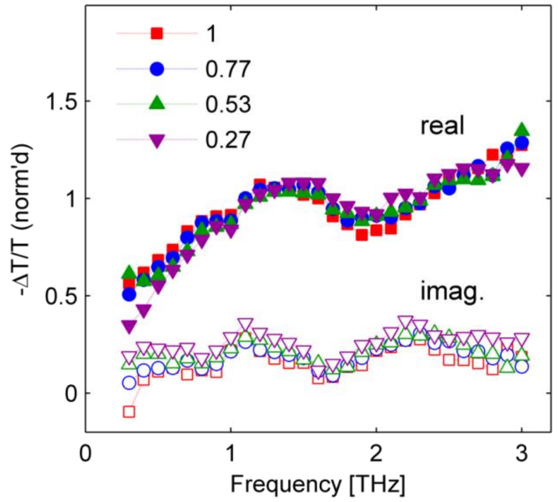

Figure S4. Photoinduced THz absorption spectra of meso-superstructured $CH_3NH_3PbI_{3-x}Cl_x$ at various electric field strengths of the THz probe pulse. (Excitation wavelength: 550 nm), fluence: 320 µJ cm^{-2}. The legend indicates the relative electric field strengths used. For comparability, all spectra are normalized by their frequency-averaged absolute value.

I. Fluence dependent time-resolved photoluminescence

To assess the diffusion length of charge carriers in these materials and its dependence on carrier density, we establish the value of the mono-molecular recombination rate. As our photoconductivity study shows, such experiments require ultra-low excitation fluences (to avoid the dominance of higher-order effects). We therefore choose time-resolved photoluminescence measurements based on time-correlated single photon counting (TCSPC) as a method allowing exceptionally high detectivity at low excitation fluences. A PicoQuant FluoTime 300 TCSPC system was used with 510 nm excitation wavelength and 1 MHz repetition rate. Counting rates were kept below 1 % of the repetition rate to avoid multi-photon events. Spectral resolution was provided by a grating-monochromator and photons counted in a hybrid photomultiplier detector. All PL measurements were performed with the sample under vacuum ($p < 10^{-5}$ mbar).

The dynamics observed with this technique may reflect a combination of both excitonic and free charge carrier populations. However, given the relatively low estimates for the exciton binding energy in these systems, excitons and charges may coexist and are probably influenced by similar mono-molecular decay channels. In addition, in the low-fluence regime, exciton formation will most likely precede charge recombination. The determined mono-molecular rates therefore represent an upper limit to what free charge carriers will experience and provide a useful reference point. We note that both exciton geminate recombination, and trap-assisted recombination (if outside the saturation regime) may lead to the observed fluence-independent, mono-molecular PL decay components. While both channels may be either radiative, or non-radiative, they will decimate the exciton and/or charge population which will be reflected in the observed PL dynamics.

Time resolved photoluminescence traces of $CH_3NH_3PbI_3$ and $CH_3NH_3PbI_{3-x}Cl_x$ were acquired for three fluences in the interval of 3 μJ cm^{-2} down to 0.03 μJ cm^{-2}, which correspond to initial absorbed photon densities between 6×10^{17} cm^{-3} and 6×10^{15} cm^{-3}. Based on the rate constants determined by THz spectroscopy we expect bimolecular recombination to only contribute significantly at the highest fluence of this range, and for lower fluences at early times before the charge density has decayed significantly. However, for the 700-ns observation window, significant charge-carrier diffusion is expected to occur, which will complicate any analysis of the decay traces in terms of higher-order decay mechanisms. In order to extract a value for the mono-molecular decay constants, we therefore focused on the longer-delay, low fluence data which will be mainly dominated by mono-molecular decay. **Figure S5** shows that indeed at longer delays the decay approaches a mono-exponential shape reflecting the monomolecular contribution; a small curvature however remains which introduces a systematic error of ~10 % to our analysis. We extract the monomolecular rate constants from the tails of the lowest fluence PL data by performing a mono-exponential decay fit to an appropriate interval (i.e. as far from the initial steep decay as possible, but still sufficiently wide). The rate constants k_1 obtained from fits shown as solid lines in Figure S5 are: $CH_3NH_3PbI_{3-x}Cl_x$: 4.9 μs^{-1}, $CH_3NH_3PbI_3$ (3:1): 15 μs^{-1} and $CH_3NH_3PbI_3$ (1:1): 14 μs^{-1}.

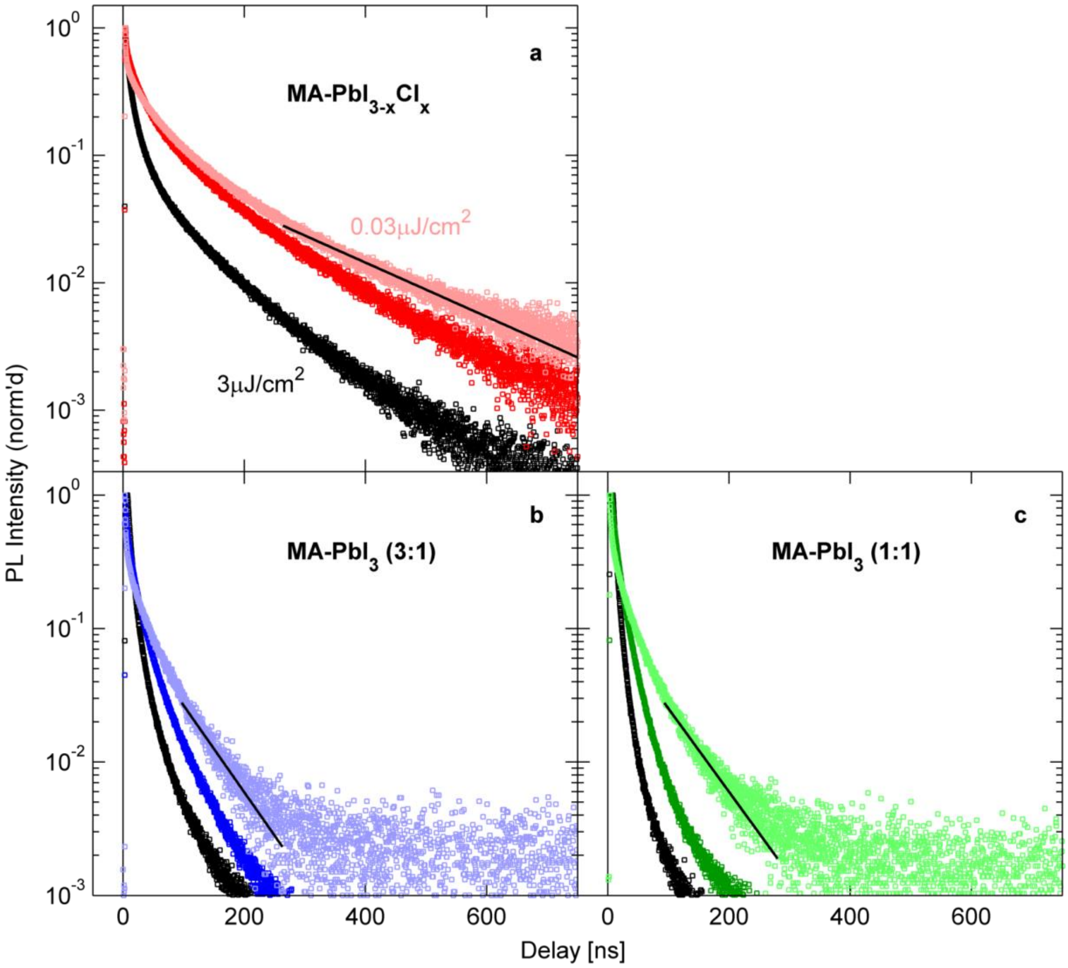

Figure S5. Time-resolved photoluminescence of $CH_3NH_3PbI_3$ and $CH_3NH_3PbI_{3-x}Cl_x$ for excitation fluences of 3 μJ cm^{-2} , 0.3 μJ cm^{-2} and 0.03 μJ cm^{-2}. (Excitation wavelength: 510 nm). A first order decay function was fitted to the tails of the lowest fluence data to extract an estimate of the monomolecular decay rate.

[1] A. Abate, T. Leijtens, S. Pathak, J. Teuscher, R. Avolio, M. E. Errico, J. Kirkpatrik, J. M. Ball, P. Docampo, I. McPherson und H. J. Snaith, *Phys. Chem. Chem. Phys.*, **2013**, *15*, 2572.

[2] P. Tiwana, P. Parkinson, M. B. Johnston, H. J. Snaith und L. M. Herz, *J. Phys. Chem. C*, **2010**, *114*, 1365-1371.

[3] H.-K. Nienhuys und V. Sundström, *Phys. Rev. B*, **2005**, *71*, 235110.

[4] a) H. Němec, P. Kužel und V. Sundström, *J. Photochem. Photobiol. A-Chem.*, **2010**, *215*, 123-139; b) R. Ulbricht, E. Hendry, J. Shan, T. F. Heinz und M. Bonn, *Rev. Mod. Phys.*, **2011**, *83*, 543-586; c) J. Lloyd-Hughes und T.-I. Jeon, *J. Infrared. Millim. Te.*, **2012**, *33*, 871-925.

[5] a) K. Lee, A. J. Heeger und Y. Cao, *Phys. Rev. B, American Physical Society*, **1993**, *48*, 14884-14891; b) N. V. Smith, *Phys. Rev. B*, **2001**, *64*, 155106; c) H. Němec, P. Kužel und V. Sundström, *Phys. Rev. B*, **2001**, *64*, 155106.
